# A liquid biopsy signature of circulating extracellular vesicles-derived RNAs predicts response to first line chemotherapy in patients with metastatic colorectal cancer

**DOI:** 10.1186/s12943-023-01875-y

**Published:** 2023-12-07

**Authors:** Ya’nan Yang, Jieyun Zhang, Wen Zhang, Yixuan Wang, Yujia Zhai, Yan Li, Wenhua Li, Jinjia Chang, Xiaoying Zhao, Mingzhu Huang, Qirong Geng, Yue Yang, Zhe Gong, Nuoya Yu, Wei Shen, Qian Li, Shenglin Huang, Weijian Guo

**Affiliations:** 1 Department of Gastrointestinal Medical Oncology, Fudan University Shanghai Cancer Center, 270 Dong-An Road, Shanghai, 200032 P. R. China; 2 Department of Oncology, Shanghai Medical College, Fudan University, Shanghai, P. R. China; 3 Department of Head & Neck Medical Oncology, Fudan University Shanghai Cancer Center, Shanghai, 200032 P. R. China; 4 Shanghai Key Laboratory of Radiation Oncology, Fudan University Shanghai Cancer Center, and Shanghai Key Laboratory of Medical Epigenetics, Institutes of Biomedical Sciences, Fudan University, Shanghai, 200032 P. R. China; 5 Department of Radiation Oncology, Shandong Cancer Hospital and Institute, Shandong First Medical University and Shandong Academy of Medical Sciences, Jinan, 250000 China; 6 Department of Colorectal Surgery, Xinhua Hospital Affiliated to Shanghai Jiao Tong University School of Medicine, Shanghai, 200092 P. R. China; 7 Department of Medical Oncology, Zhongshan Hospital, Fudan University, No.180, Fenglin Road, Shanghai, 200032 P. R. China

**Keywords:** Colorectal cancer, Extracellular vesicles, Signature, Prediction, Chemotherapy

## Abstract

**Background:**

Colorectal cancer (CRC) is one of the most threatening tumors in the world, and chemotherapy remains dominant in the treatment of metastatic CRC (mCRC) patients. The purpose of this study was to develop a biomarker panel to predict the response of the first line chemotherapy in mCRC patients.

**Methods:**

Totally 190 mCRC patients treated with FOLFOX or XEOLX chemotherapy in 3 different institutions were included. We extracted the plasma extracellular vesicle (EV) RNA, performed RNA sequencing, constructed a model and generated a signature through shrinking the number of variables by the random forest algorithm and the least absolute shrinkage and selection operator (LASSO) algorithm in the training cohort (*n* = 80). We validated it in an internal validation cohort (*n* = 62) and a prospective external validation cohort (*n* = 48).

**Results:**

We established a signature consisted of 22 EV RNAs which could identify responders, and the area under the receiver operating characteristic curve (AUC) values was 0.986, 0.821, and 0.816 in the training, internal validation, and external validation cohort respectively. The signature could also identify the progression-free survival (PFS) and overall survival (OS). Besides, we constructed a 7-gene signature which could predict tumor response to first-line oxaliplatin-containing chemotherapy and simultaneously resistance to second-line irinotecan-containing chemotherapy.

**Conclusions:**

The study was first to develop a signature of EV-derived RNAs to predict the response of the first line chemotherapy in mCRC with high accuracy using a non-invasive approach, indicating that the signature could help to select the optimal regimen for mCRC patients.

**Supplementary Information:**

The online version contains supplementary material available at 10.1186/s12943-023-01875-y.

## Introduction

Colorectal cancer (CRC) is a common malignant tumor. According to the latest data revealed by GLOBOCAN 2020, the global incidence of CRC in 2020 ranks third in the world [1]. According to statistics, 20% of patients already have distant metastasis when diagnosed with CRC, and approximately 50% of patients diagnosed with limited-stage CRC will later develop metastasis [2]. Despite advances in early detection and management of multiple treatment modalities, the prognosis for metastatic CRC (mCRC) remains poor, with a 5-year survival rate of less than 20% [3].

Nowadays, the major therapeutic regimens are systematic chemotherapy with or without target therapy agents [2]. Meanwhile, immune checkpoint inhibitors have also made great progress in CRC treatment [4]. It is revealed that patients with deficient mismatch repair (dMMR) or microsatellite instability high (MSI-H) tumors could benefit more from immunotherapy than chemotherapy [5]. Unfortunately, majorities of CRC patients had microsatellite stable (MSS) tumors, for whom immunotherapy was almost ineffective [6, 7]. Therefore, chemotherapy remains dominant in the treatment of mCRC patients, and in general, either oxaliplatin-based regimens (FOLFOX or CAPOX) or irinotecan-based regimens (FOLFIRI) could be the major choice for first-line chemotherapy [3].

Recently, there is a growing emphasis on precision medicine. The rapid development of next-generation sequence (NGS) provides technical support for precision medicine, and more and more studies are applying this technology to find markers related to tumor diagnosis, treatment or prognosis and so on [8, 9]. Many researches present biomarkers for immunotherapy and targeted therapy [10, 11], but rare for chemotherapy. It is convenient and safe to obtain the genetic information needed for diagnosis or treatment through blood collection, and it has been presented that extracellular vesicles (EVs, consisting of exosomes and microvesicles) in the circulatory system are an important source of diagnostic molecular markers. For example, a research has developed a diagnostic signature for pancreatic ductal adenocarcinoma through plasma exosome RNA sequence [12]. In addition, a recent study has established a plasma EV long RNA signature, which has potential clinical value for SCLC diagnosis and treatment [13]. Given that, we focused our attention on the EVs which contain cargos like RNA and proteins [14]. A lately published paper has presented us with an exosome‑derived signature that can predict the therapeutic efficacy of neoadjuvant chemotherapy in patients with advanced gastric cancer [15]. However, there are few articles that focus on predicting the efficacy of first-line chemotherapy through plasma EVs examination in mCRC.

In this study, we performed the EVs RNA sequencing on plasma samples collected from a total of 190 mCRC patients, looking forward to establishing an EV-based RNAs signature to predict the objective response rate (ORR) of first line chemotherapy in mCRC.

## Results and discussion

### Establishment and validation of the EV-derived RNAs signature for predicting ORR of first-line chemotherapy in mCRC

Totally 190 patients were finally included in the analysis and among which, 142 participants were recruited from Fudan University Shanghai Cancer Center (FUSCC), and 48 patients were recruited from Zhongshan hospital, Fudan University and Xinhua Hospital Affiliated to Shanghai Jiao Tong University School of Medicine. Among the 142 patients treated at FUSCC, we randomly divided the patients treated with single chemotherapy (*n* = 120) into the training set and internal validation set proportionally (2:1), and those with target therapy are put into the internal validation set, so training cohort (*n* = 80) and internal validation cohort (*n* = 62). Patients treated in Zhongshan hospital and Xinhua hospital were included in external validation set, the samples in which were collected prospectively. The clinicopathological characteristics of the patients were shown in Supplement Table 1, indicating that the three cohort were balanced well in terms of gender, age, tumor site, pathological type, degree of differentiation, and the proportion of mutations in RAS or BRAF.

The mCRC patients were treated with FOLFOX (oxaliplatin 85 mg/m^2^, Leucovorin 400 mg/m^2^, and fluorouracil 400 mg/m^2^ intravenously on day 1, followed by a 46 h continuous infusion of fluorouracil 2400 mg/m^2^; repeated every two weeks) or XEOLX (oxaliplatin 130 mg/m^2^ intravenously on day 1; capecitabine 1000 mg / m^2^ p.o. bid from day 1 to day 14; repeated every three weeks) chemotherapy with or without anti-VEGF or anti-EGFR antibodies as first line treatment. The radiologic evaluations of tumor responses were performed every 2–3 cycles during systematic chemotherapy and every 2 months during the maintenance therapy. The tumor response was assessed according to RECIST 1.1 criteria. The follow-up in our study was aimed at PFS (defined as the time from treatment to first progression or death due to various causes) and OS (defined as the time from treatment to death).

**Fig. 1:**
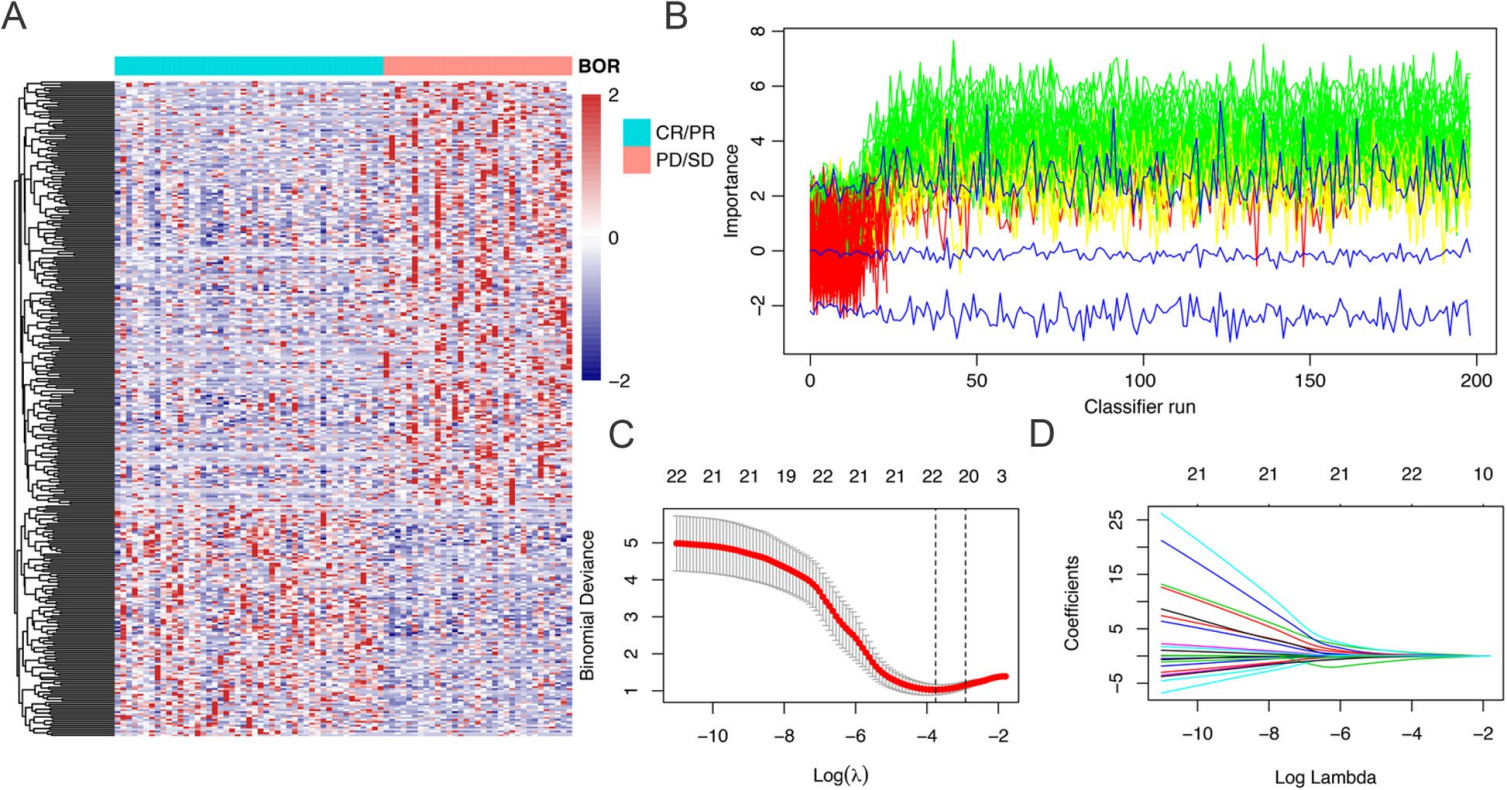
Construction of the 22-gene signature. **A** Heatmap of 340 genes which were differentially expressed between different BOR (patients with CR + PR versus PD + SD) in the training cohort. Each column represents an individual sample, and each row represents a gene. *P* < 0.05. *The TPM values centered and scaled in the row direction by R package ‘pheatmap’. **B** The ranking and selection of BOR associated variates using the random forest algorithm. **C**, **D** The most robust predictive 22 genes were identified using LASSO-logistic algorithm

**Fig. 2:**
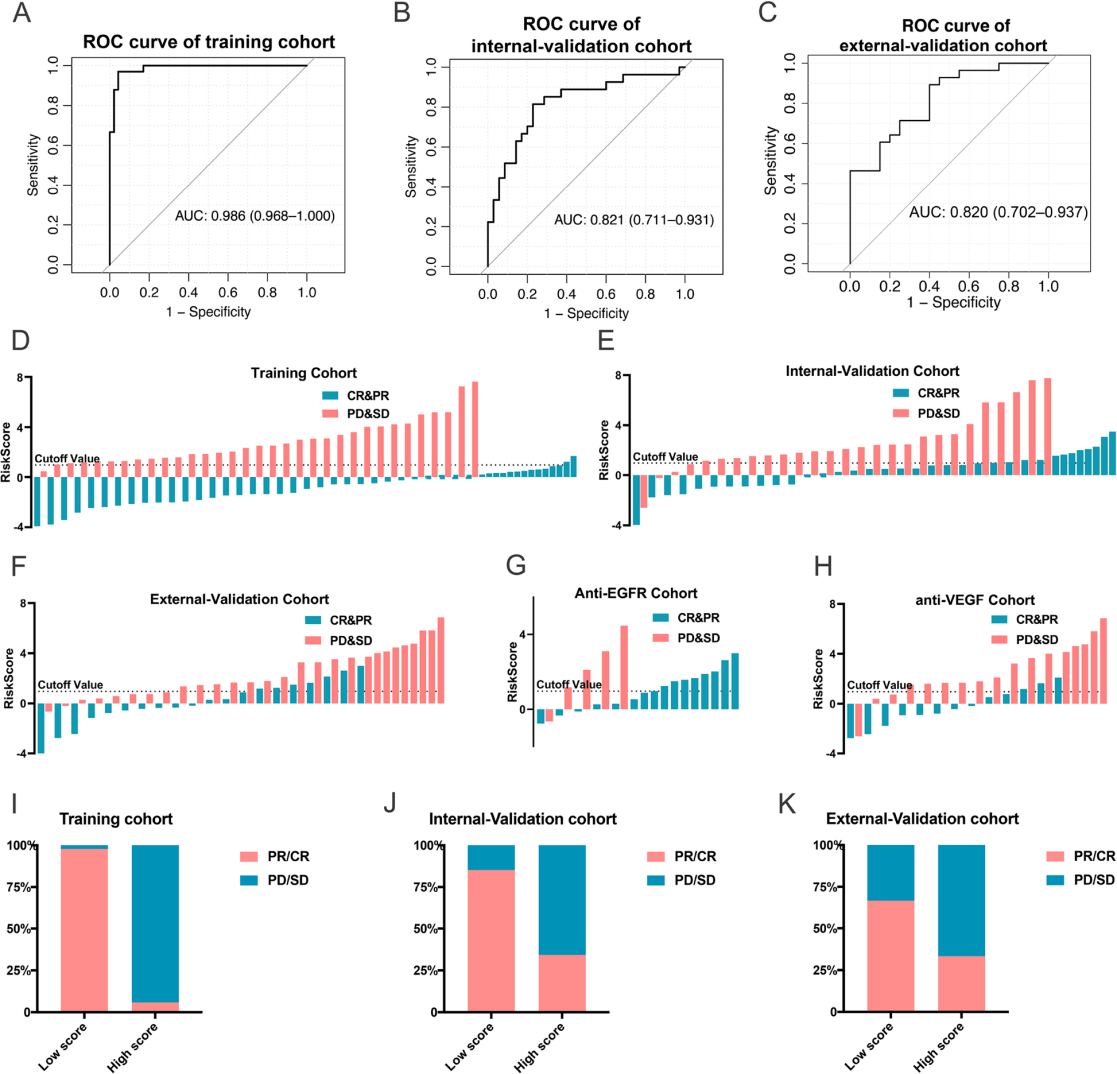
Predictive ability of the 22-gene signature in different cohort. The ROC analyses illustrated the predictive ability of this 22-gene model to identify responders in the training set (**A**), internal validation set (**B**), and external validation set (**C**); The scores of enrolled patients in different cohorts which were calculated based on the 22-gene signature: the training cohort (**D**), the internal validation cohort (**E**), the external validation cohort (**F**), the cohort combined chemotherapy with anti-EGFR (**G**), the cohort combined chemotherapy with anti-VEGF (**H**). Patients with lower risk score would more likely obtain tumor remission in training group (**I**), internal validation group (**J**) and external validation group (**K**)

We extracted the RNAs of isolated EVs from 190 patients and performed the RNA sequence referring to the methodological part of the previous study published by the same laboratory personnel [12]. About 58132 genes were finally detected in each sample, including mRNAs, non-coding RNAs and so on. We randomly selected eight genes and examined their expression in five randomized patients by real-time quantitative reverse transcription polymerase chain reaction (RT-PCR), and the results were in approximately 95% agreement with the sequencing data. We divided the patients in the training set into two subgroups according to the best of response (BOR) during the first-line chemotherapy, which were identified as response (complete response, CR and partial response, PR) group and non-response (stable disease, SD and progressive disease, PD) group. None of the clinicopathologic factors were significantly related to ORR, as shown in Supplement Table 2, indicating that the clinicopathologic factors could not predict ORR. Then we got 340 differentially expressed genes between the two groups. Figure 1A was the heat map of 340 different genes between the two groups in training set (*p* < 0.05). We then applied the random forest algorithm using R package ‘varSelRF’ (10.1186/1471-2105-8-328) and the least absolute shrinkage and selection operator (LASSO) method for feature ranking to find and shrink the significant variables associated with BOR, and as shown in Fig. 1B-D, the green color represented the variables we may be probably interested. Finally, 22 markers (GOLPH3L, RPL21P1, ANKRD20A17P, CCL2, EPS8L1, CUBN, PODXL, ALG9, TLR5, CLK4, PRICKLE2, HMGB1P24, ME3, RPL13P12, MIB2, SLC5A6, NUDT19, ELMOD3, PGAM5, ERICH6-AS1, BACE1, MYL12BP1) were selected to establish a lasso-logistic model using R package ‘glmnet’ (10.18637/jss.v033.i01) for predicting the response of first-line chemotherapy in mCRC patients. The 22-gene signature distinguished patients with respondence from patients with non-respondence, with an AUC of 0·986 (95%CI: 0·968 to 1·000) in the training set (Fig. 2A). Then we applied the signature to the internal validation set and the AUC was 0·821 (95%CI: 0·711 to 0·931) (Fig. 2B). To further confirm the prediction ability of the 22-gene signature on first-line chemotherapy in mCRC patients, we applied the signature to a prospective external validation set, and the AUC was 0·820 (95%CI: 0·702 to 0·937) (Fig. 2C). Supplement Table 3 demonstrates the percentage of ORR in patients with different risk score in varieties of cohorts. The ability of the 22-gene signature to predict the response to first-line chemotherapy in the training set was visually exhibited in the Fig. 2D and I, and patients with lower risk-score according to the model would more likely obtain a BOR of CR or PR (*p* < 0·05). The signature was applied in the internal validation set and external validation set and got similar results (Fig. 2E, J, F, K). Meanwhile, the signature was applied in patients treated with chemotherapy combined with anti-VEGF target therapy among the internal and external validation cohorts, and we found that the 22-gene signature could also well distinguish patients with different BOR (*p* < 0·05), as shown in Fig. 2H. Likewise, the signature was applied in patients treated with chemotherapy combined with anti-EGFR target therapy among the two validation cohorts, and we found that compared with patients with high-risk score, the ORR in the patients with low-risk score was higher but the difference between them was not statistically significant, which may be related to the fact that anti-EGFR antibodies can modify or reverse chemotherapy resistance (Supplement Table 3, Fig. 2G).

### The 22-gene signature was identified as a predictor of PFS and OS in mCRC patients treated with first-line chemotherapy

We identified the prognostic value of the 22-gene signature both in training cohort and validation cohorts using R package ‘survival’(https://CRAN.R-project.org/package=survival). The patients of the training and validation cohorts were both divided into two subgroups, the low-risk score subgroup and the high-risk score subgroup, according to the cutoff value obtained from the Receiver operating characteristic (ROC) curve. Compared with patients with high-risk score, patients with low-risk score had longer OS and PFS in training cohort (*p* = 0·00016 and *p* < 0·0001 respectively), as shown in Supplement Fig. 1A-B, which was the same as the results in the whole validation cohort (Supplement Fig. 1C-D, *p* = 0·02 and *p* = 0·047 respectively).

### GSEA analysis for the gene in the 22-gene signature

We observed the expression of the genes in the 22-gene model in the TCGA database, and the differentially expressed genes in the cancer versus the adjacent normal tissues were subjected to signaling pathway analysis through GSEA software (10.1073/pnas.0506580102). Genes would be selected with adjustment *P* < 0·05 and a change greater than 1·5-fold after paired DEseq2 analysis (10.1186/s13059-014-0550-8). The following genes of the 22-gene model met the requirements: CCL2 (high in adjacent normal tissues), PODXL (high in cancer), TLR5 (high in adjacent normal tissues), PRICKLE2 (high in adjacent normal tissues), SLC5A6 (high in cancer), PGAM5 (high in cancer). Fifty key HALLMARK pathways were analyzed, the upregulated pathways in patients with a higher gene expression were shown in Supplement Fig. 2. Down-regulations of CCL2 enriched the MYC associated signaling pathway, and up-regulation of PGAM5 or down-regulation of PODXL enriched the oxidative phosphorylation pathway. Besides, higher PRICKLE2 expression could enrich the inflammatory pathway and lower TLR5 expression always enriched the DNA repair pathway. The specific mechanism deserves further in-depth study, and the relevant molecules we screened in this study may also provide new targets for the exploration of the mechanism associated with chemo-resistance in order to further overcome drug resistance.

### Establishment of an EV-derived 7-gene signature to predict the response of oxaliplatin contained first-line chemotherapy and irinotecan contained second-line chemotherapy in mCRC

To construct a model through which we can predict the response of both oxaliplatin contained chemotherapy and irinotecan contained chemotherapy, we selected patients with second-line treatment data on the use of irinotecan-containing regimens. The ORR was applied to assess the response of first-line chemotherapy while in the assessment of second-line chemotherapy, we selected the disease control rate (DCR) because of low ORR in second-line treatment. We screened the molecules related with good response to first-line oxaliplatin contained regimens and poor response to second-line irinotecan contained-regimens, and vice versa. We then applied the LASSO method for feature ranking (Supplement Fig. 3A-B) and finally established a 7-gene signature which containing CACNG6, PCSK5, CCL2, TBC1D24, NUBPL, PIK3R4, AL513550.1. The 7-gene signature predicted well in the response of oxaliplatin contained chemotherapy, as shown in Supplement Fig. 3E, with an AUC of 0·835 (95%CI: 0·737 to 0·932); moreover, the prediction of irinotecan-contained chemotherapy was exhibited in Supplement Fig. 3G, with an AUC of 0·793 (95%CI: 0·654 to 0·931). Each patient could obtain a risk score according to the 7-gene signature, and patients with lower risk score would more likely obtained tumor remission (CR or PR) in the first-line oxaliplatin contained chemotherapy, as shown in Supplement Fig. 3C,F, while in the second-line irinotecan contained chemotherapy, patients with lower risk score tend to have greater possibility to suffer PD, which was shown in Supplement Fig. 3D,H. Benefit from this, we may apply this model to the selection of first-line treatment options, helping us to choose the best one more accurately. The limitation is that the number of cases in this model is small and the irinotecan containing regimen is used as the second line treatment, so future validation is worthwhile in a larger sample and in patients treated with irinotecan containing regimens as first-line chemotherapy.

## Conclusions

Our study is the first study to apply EV RNA sequencing technology and successfully predict the response of palliative treatment in mCRC patients by constructing a model in a training set and validating it in an internal and external validation set. We developed a 22 EV-based RNAs signature with response prediction ability and it could well stratify the mCRC patients to the oxaliplatin-based chemotherapy sensitive group and insensitive group before first-line chemotherapy. Then clinicians could select the more suitable regimens for patients to avoid delaying the treatment opportunity. Thus, individualized treatment would be achieved, which is expected to improve the chances of achieving tumor remission, especially for patients treated with conversion therapy, or with large tumor loads.

### Supplementary Information

**Additional file 1: Supplement Fig. 1.** Survival curves for patients with different risk score according to the 22-gene signature  A the survival curves of OS for patients in training cohort; B the survival curves of PFS for patients in training cohort; C the survival curves of OS for patients in the whole validation cohort; D the survival curves of PFS for patients in the whole validation cohort. **Supplement Fig. 2.** GSEA analysis for the gene in the 22-gene signature which were differentially expressed in the cancer versus the adjacent normal tissues in the TCGA database. **Supplement Fig. 3.** Construction and the predictive ability of the 7-gene signature  (A-B) The random forest algorithm and LASSO method for feature ranking were utilized to establish a 7-gene signature; (E) The 7-gene signature predicted well in the efficacy of oxaliplatin contained chemotherapy; (G) the ROC curves for the prediction of irinotecan-contained chemotherapy; (C, F) patients with lower risk score would more likely obtained tumor remission in the first-line oxaliplatin contained chemotherapy; (D, H) patients with lower risk score tend to have greater possibility to suffer PD in the second-line irinotecan contained chemotherapy. **Supplement Table 1.** The clinicopathological characteristics of the mCRC patients enrolled. **Supplement Table 2.** The clinicopathological characteristics of patients with different ORR in the training cohort. **Supplement Table 3.** The ORR of patients with different risk score in varieties of cohorts. 

## Data Availability

The datasets supporting the conclusions of this article are included within the article.

## Abbreviations

CRC: Colorectal cancer
mCRC: Metastatic CRC
LASSO: Least absolute shrinkage and selection operator
AUC: Area under the curve
OS: Overall survival
PFS: Progression-free survival
dMMR: Deficient mismatch repair
MSI-H: Microsatellite instability-high
MSS: Microsatellite stable
NGS: Next-generation sequence
ctDNA: Circulating tumor DNA
EVs: Extracellular vesicles
RECIST: Response Evaluation Criteria in Solid Tumors
FUSCC: Fudan University Shanghai Cancer Center
TEM: Transmission electron microscopy
BOR: Best of response
SVM: Support Vector Machine
OBB: Out-of-bag
DCR: Disease control rate
ORR: Objective response rate
PBMC: Peripheral blood mononuclear cell
ROC: Receiver operating characteristic
RT- PCR: Real-time quantitative reverse transcription polymerase chain reaction
TCGA: The Cancer Genome Atlas
