# Supplementary material for: A liquid biopsy signature of circulating extracellular vesicles-derived RNAs predicts response to first line chemotherapy in patients with metastatic colorectal cancer
Source: Mol Cancer. 2023 Dec 7;22:199. doi: 10.1186/s12943-023-01875-y (PMC10701920; doi:10.1186/s12943-023-01875-y)
Supplement: Supplementary file 1 — Additional file 1: Supplement Fig. 1. Survival curves for patients with different risk score according to the 22-gene signature A the survival curves of OS for patients in training cohort; B the survival curves of PFS for patients in training cohort; C the survival curves of OS for patients in the whole validation cohort; D the survival curves of PFS for patients in the whole validation cohort. Supplement Fig. 2. GSEA analysis for the gene in the 22-gene signature which were differentially expressed in the cancer versus the adjacent normal tissues in the TCGA database. Supplement Fig. 3. Construction and the predictive ability of the 7-gene signature (A-B) The random forest algorithm and LASSO method for feature ranking were utilized to establish a 7-gene signature; (E) The 7-gene signature predicted well in the efficacy of oxaliplatin contained chemotherapy; (G) the ROC curves for the prediction of irinotecan-contained chemotherapy; (C, F) patients with lower risk score would more likely obtained tumor remission in the first-line oxaliplatin contained chemotherapy; (D, H) patients with lower risk score tend to have greater possibility to suffer PD in the second-line irinotecan contained chemotherapy. Supplement Table 1. The clinicopathological characteristics of the mCRC patients enrolled. Supplement Table 2. The clinicopathological characteristics of patients with different ORR in the training cohort. Supplement Table 3. The ORR of patients with different risk score in varieties of cohorts. [file 12943_2023_1875_MOESM1_ESM.docx]

**Supplement** **Fig. 1** Survival curves for patients with different risk score according to the 22-gene signature A the survival curves of OS for patients in training cohort; B the survival curves of PFS for patients in training cohort; C the survival curves of OS for patients in the whole validation cohort; D the survival curves of PFS for patients in the whole validation cohort.

**Supplement Fig. 2** GSEA analysis for the gene in the 22-gene signature which were differentially expressed in the cancer versus the adjacent normal tissues in the TCGA database.


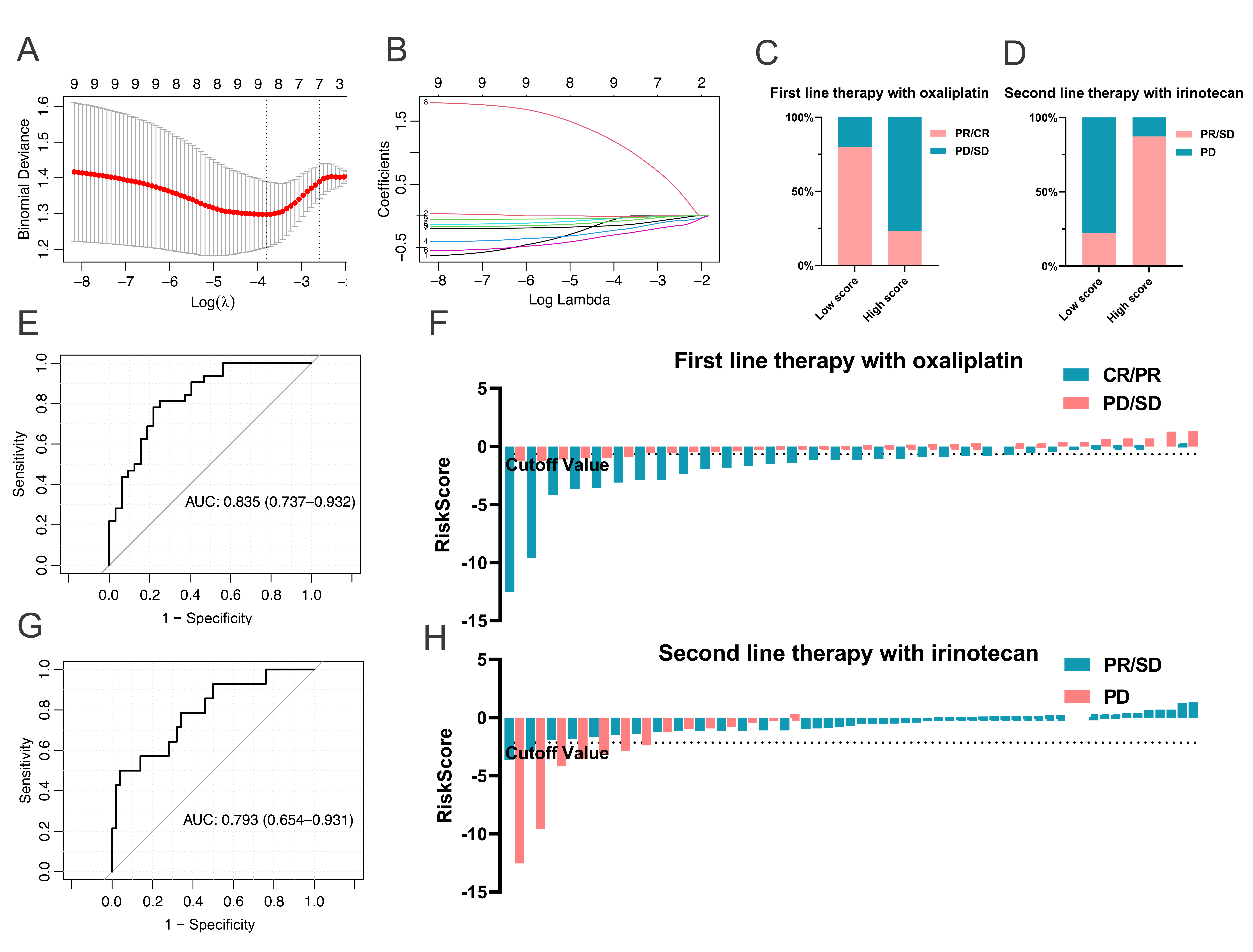


**Supplement Fig. 3** Construction and the predictive ability of the 7-gene signature (A-B) The random forest algorithm and LASSO method for feature ranking were utilized to establish a 7-gene signature; (E) The 7-gene signature predicted well in the efficacy of oxaliplatin contained chemotherapy; (G) the ROC curves for the prediction of irinotecan-contained chemotherapy; (C, F) patients with lower risk score would more likely obtained tumor remission in the first-line oxaliplatin contained chemotherapy; (D, H) patients with lower risk score tend to have greater possibility to suffer PD in the second-line irinotecan contained chemotherapy.

**Supplement Table 1 The clinicopathological characteristics of the mCRC patients enrolled.**

|  | **Training cohort** | **Internal validation cohort** | **External validation cohort** | **Total** | **P value** |
| --- | --- | --- | --- | --- | --- |
| **gender** |  |  |  |  | 0.09 |
| Female | 28 | 28 | 12 | 68 |  |
| Male | 52 | 34 | 36 | 122 |  |
| **age** |  |  |  |  | 0.034 |
| <50 | 13 | 18 | 5 | 36 |  |
| ≥50 | 67 | 44 | 43 | 154 |  |
| **site** |  |  |  |  | 0.512 |
| Right half | 23 | 20 | 14 | 57 |  |
| Left half | 56 | 42 | 32 | 130 |  |
| Both | 1 | 0 | 2 | 3 |  |
| - **Pathology** |  |  |  |  | 0.682 |
| - adenocarcinoma | 76 | 56 | 47 | 179 |  |
| - signet-ring cell carcinoma | 1 | 2 | 1 | 4 |  |
| - mucinous adenocarcinoma | 2 | 3 | 0 | 5 |  |
| NA | 1 | 1 | 0 | 2 |  |
| **Differentiation** |  |  |  |  | 0.054 |
| Low | 13 | 10 | 13 | 36 |  |
| Middle | 40 | 24 | 22 | 86 |  |
| High | 4 | 0 | 0 | 4 |  |
| NA | 23 | 28 | 13 | 64 |  |
| **MMR** |  |  |  |  | 0.003 |
| dMMR/MSI-H | 4 | 0 | 1 | 5 |  |
| pMMR/MSS | 54 | 43 | 45 | 142 |  |
| NA | 22 | 19 | 2 | 43 |  |
| **RAS/BRAF** |  |  |  |  | 0.177 |
| Mutation | 36 | 28 | 21 | 85 |  |
| Wildtype | 27 | 24 | 24 | 75 |  |
| NA | 17 | 10 | 3 | 30 |  |
| **Therapy** |  |  |  |  | <0.001 |
| Chemotherapy | 80 | 40 | 20 | 140 |  |
| Chemotherapy +anti-VEGF | 0 | 13 | 17 | 30 |  |
| Chemotherapy +anti-EGFR | 0 | 9 | 11 | 20 |  |
|  |  |  |  |  |  |

**Supplement Table 2 The clinicopathological characteristics of patients with different ORR in the training cohort.**

|  | **PR/CR** | **%** | **SD/PD** | **%** | **P value** |
| --- | --- | --- | --- | --- | --- |
| **gender** |  |  |  |  | 0.83 |
| Female | 16 | 34.04 | 12 | 36.36 |  |
| Male | 31 | 65.96 | 21 | 63.64 |  |
| **age** |  |  |  |  | 0.823 |
| <50 | 8 | 17.02 | 5 | 15.15 |  |
| ≥50 | 39 | 82.98 | 28 | 84.85 |  |
| **site** |  |  |  |  | 0.458 |
| Right half | 13 | 27.66 | 10 | 30.3 |  |
| Left half | 34 | 72.34 | 22 | 66.67 |  |
| Both | 0 | 0 | 1 | 3.03 |  |
| - **Pathology** |  |  |  |  | 0.235 |
| - adenocarcinoma | 45 | 95.74 | 31 | 93.94 |  |
| - signet-ring cell carcinoma | 0 | 0 | 1 | 3.03 |  |
| - mucinous adenocarcinoma | 2 | 4.26 | 0 | 0 |  |
| NA | 0 | 0 | 1 | 3.03 |  |
| **Differentiation** |  |  |  |  | 0.298 |
| Low | 7 | 14.89 | 6 | 18.18 |  |
| Middle | 27 | 57.45 | 13 | 39.39 |  |
| High | 1 | 2.13 | 3 | 9.09 |  |
| NA | 12 | 25.53 | 11 | 33.33 |  |
| **MMR** |  |  |  |  | 0.936 |
| dMMR/MSI-H | 2 | 4.26 | 2 | 6.06 |  |
| pMMR/MSS | 32 | 68.09 | 22 | 66.67 |  |
| NA | 13 | 27.66 | 9 | 27.27 |  |
| **RAS/BRAF** |  |  |  |  | 0.804 |
| Mutation | 21 | 44.68 | 15 | 45.45 |  |
| Wildtype | 17 | 36.17 | 10 | 30.3 |  |
| NA | 9 | 19.15 | 8 | 24.24 |  |

**Supplement Table 3 The ORR of patients with different risk score in varieties of cohorts.**

|  | **ORR%** | | **P value** |
| --- | --- | --- | --- |
|  | **Low-Risk** | **High-Risk** |  |
| **Training cohort** | 97.83 | 5.88 | 0.000 |
| **Internal Validation** | 85.19 | 34.29 | 0.000 |
| **External Validation** | 61.90 | 25.93 | 0.000 |
| **Validation Chemotherapy only** | 70.37 | 24.24 | 0.000 |
| **Validation Chemotherapy+anti-EGFR** | 87.50 | 66.67 | 0.603 |
| **Validation Chemotherapy+anti-VEGF** | 76.92 | 17.65 | 0.002 |
